# Acetate of Lead for Granular Lids

**Published:** 1851-03

**Authors:** 


*Acetate of Lead for Granular Lids.*—Dr. Cunier, of Brussels, has recently published the successful results of the practice of Dr. Buys in granular lid. His treatment consisted in the application of the acetate of lead to the palpebral conjunctiva, instead of the nitrate of silver commonly used. Dr. Buys takes neutral acetate of lead, reduced to an impalpable powder, dips a moistened pencil into it, and takes up about a grain, or a grain and a half of the salt, which is enough for one lid. When the lid is touched, it should be kept everted until the tears have dissolved the acetate, and those portions of the salt which escape solution should be taken off with the pencil. On the upper lid the tears will be insufficient to dissolve the salt, and moisture must be applied to it from the angles of the eye. As soon as the acetate is dissolved by the tears, and has penetrated the tissues, the latter are noticed to contract powerfully, and a very remarkable phenomenon takes place; for the granulations, and the grooves between them, disappear at once, if they be of a moderate size, and the conjunctiva looks smooth and uniform as long as it remains exposed to the air; but the shining, white color produced by the lead is perceived only when the lid comes again into contact with the globe of the eye. When the granulations and furrows between them are large, two or three applications are necessary to obtain smoothness; one, however, is sufficient when they are not of great size. These applications produce more burning and astriction than pain, and when the lid is replaced, hot and whitish tears escape, the flow of which may last from a few seconds to two or three minutes; it rarely lasts longer, and the eye can then be kept open. When the granulations are of an indolent character, the slight injection of the ocular conjunctiva, which occurs on the application of the lead, goes off rapidly; if, on the other hand, the cornea be vascular, the vessels become smaller soon after the cauterization of the lids. Slight œdema of the palpebræ may follow, but in twenty-four or forty-eight hours it wholly disappears; a dry compress rubbed with camphor will hasten resolution, and likewise lessen the burning sensation, if the latter happen to persist. One lid only should be touched at a time, and not again be interfered with, unless all trace of the salt is removed. Dr. Buys has used the lead in a great many cases of contagious ophthalmia among the military, and Dr. Cunier in acute and chronic catarrhal ophthalmia; the results seem to have been more satisfactory in the latter variety, as also in scrofulous affections of the eye, vascular cornea, and ulcers of that membrane. The mineral has likewise succeeded in cutting short incipient inflammation of the organ.—*Half Yearly Abstract of the Medical Sciences.*

